# Massive Bilateral Pulmonary Embolism Presenting as Isolated Abdominal Pain: An Atypical Presentation and Incidental Detection in Dual Immune-Mediated Disease

**DOI:** 10.7759/cureus.105063

**Published:** 2026-03-11

**Authors:** Rani Marhaba, Manjari Sriparna, Mohamed Hamzane, Sonya Kalani, Shannon Blaney

**Affiliations:** 1 Internal Medicine, Montefiore Medical Center, Bronx, USA

**Keywords:** ct abdomen, hypotension, mm: multiple myeloma, myasthenia gravis (mg), pulmonary emboli, rare cause of acute abdominal pain

## Abstract

Pulmonary embolism (PE) is a potentially life-threatening condition that classically presents with chest pain, tachycardia, and dyspnea. Atypical manifestations such as gastrointestinal symptoms may occur, particularly in elderly patients with multiple comorbidities that can potentially skew and delay diagnosis. We report the case of a 79-year-old woman with a history of multiple myeloma and myasthenia gravis who presented with acute-onset abdominal pain with nausea and vomiting. Computed tomography (CT) of the abdomen and pelvis was obtained to evaluate suspected intra-abdominal pathology that incidentally detected pulmonary arterial filling defects in the right lower lobe of the lung. Subsequent CT pulmonary angiography confirmed extensive bilateral pulmonary emboli. The patient was urgently transferred to a tertiary care center for mechanical pulmonary thrombectomy with successful hemodynamic stabilization. This case highlights an atypical presentation of life-threatening PE that presented predominantly with gastrointestinal symptoms and was incidentally identified on abdominal imaging. Clinicians should maintain a high index of suspicion for PE in patients with unexplained gastrointestinal complaints, particularly in the setting of comorbidities such as malignancy and autoimmune disease that may mimic symptoms.

## Introduction

Pulmonary embolism (PE) is a common yet potentially fatal cardiovascular condition with a broad spectrum of clinical presentations. Classic symptoms include acute dyspnea, pleuritic chest pain, hypoxia, and tachycardia. However, atypical and non-specific manifestations have been noted in the literature, particularly among elderly patients and those with multiple comorbidities, including malignancy [1]. Such presentations may delay diagnosis and can contribute to increased morbidity and mortality.

Gastrointestinal symptoms, including abdominal pain, nausea, and vomiting, are uncommon yet clinically significant primary presentations of PE and are often misattributed to intra-abdominal pathology. In the absence of chest pain or overt respiratory distress, these symptoms may divert initial evaluation away from acute cardiopulmonary causes.

We present the case of a patient predominantly presenting with abdominal pain with concurrent hypoxia and hypotension who was found to have an incidental PE on abdominal imaging. Further evaluation revealed high-risk, extensive bilateral PE requiring urgent thrombectomy for eventual clinical stabilization. 

## Case presentation

A 79-year-old woman with a past medical history significant for multiple myeloma (MM), myasthenia gravis (MG), hypertension, and type 2 diabetes mellitus presented to the emergency department with acute-onset abdominal pain, nausea, and vomiting. She had been in her usual state of health until the sudden onset of symptoms at home. She denied chest pain.

On arrival, the patient was found to be hypotensive and hypoxic, with concern for impending hemodynamic instability (Table 1). Physical examination was significant for epigastric discomfort with guarding without rebound tenderness. Bowel sounds were reported to be hypoactive. Her cardiovascular examination was regular with no obvious murmurs or gallops. Lung sounds were clear to auscultation bilaterally. 

**Table 1:** Vital signs on arrival to the emergency room Interpretation: Vital signs were notable for hypotension with hypoxia.

Vital sign	Value
Heart rate	91 beats/min
Blood pressure	97/58 mmHg
Respiratory rate	17 breaths/min
Oxygen saturation	87% on room air
Temperature	97.9°F

Laboratory studies were urgently collected (Table 2). Chest radiograph and electrocardiogram demonstrated no acute cardiopulmonary abnormalities.

**Table 2:** Initial significant laboratory results *Hemoglobin was 13.5 g/dL one month before presentation.

Laboratory test	Result	Reference range
Troponin I	0.73 ng/mL	<0.04 ng/mL
Lactic acid	4.5 mmol/L	0.5-2.2 mmol/L
Glucose	333 mg/dL	70-140 mg/dL
Total bilirubin	2.1 mg/dL	0.2-1.2 mg/dL
Potassium	3.2 mmol/L	3.5-5.1 mmol/L
Blood urea nitrogen	7 mg/dL	7-20 mg/dL
Creatinine	0.55 mg/dL	0.6-1.3 mg/dL
Albumin	2.7 g/dL	3.5-5.0 g/dL
Total protein	4.0 g/dL	6.0-8.3 g/dL
White blood cell count	5.4 × 10³/µL	4.0–11.0 × 10³/µL
Hemoglobin*	9.0 g/dL*	12.0–16.0 g/dL
Platelet count	135 × 10³/µL	150–400 × 10³/µL

**Figure 1:**
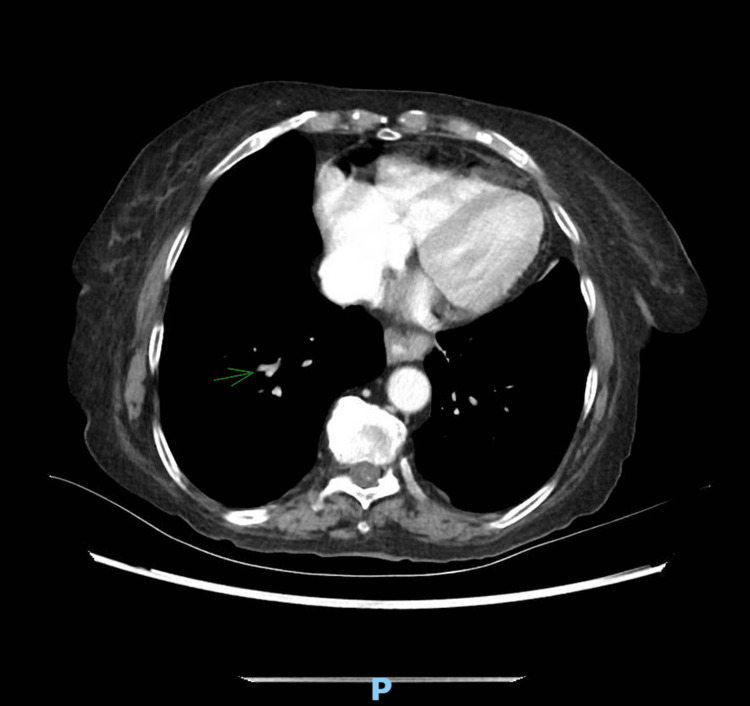
CT abdomen pelvis with incidental hypodensity observed on the right lower lung, suspicious for PE (green arrow) CT, computed tomography; PE, pulmonary embolism.

The patient was promptly started on broad-spectrum antibiotics, given initial concern for an intra-abdominal process. The patient was rushed to obtain a computed tomography (CT) of the abdomen and pelvis with contrast, which was unrevealing for acute pathology. However, the study incidentally revealed a partially visualized hypodensity at the base of the lungs within the right lower lobe segmental pulmonary artery (Figure 1), suspicious for a PE.

**Figure 2:**
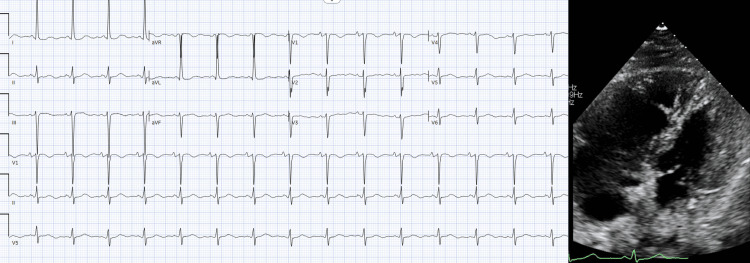
Electocardiogram and transthoracic echocardiography (TTE) The electrocardiogram was unrevealing for any acute cardiopulmonary abnormalities. However, TTE revealed significant right ventricular dilatation and dysfunction - a new finding compared with the previous TTE approximately a year before this study.

Given this initial finding, a bedside transthoracic echocardiogram (TTE) was obtained to evaluate for right heart strain, which revealed "McConnell's sign" with right ventricular dilation with reduced function (Figure 2). Immediate CT pulmonary angiography was obtained, demonstrating extensive bilateral pulmonary emboli involving branches of the right lower lobe, left lower lobe, and left upper lobe, with smaller emboli in the right upper lobe.

The patient was promptly initiated on anticoagulation with a heparin infusion and a norepinephrine drip for hemodynamic support. Given the degree of her thrombi burden across both lungs with evidence of acute cardiac strain, she was urgently transferred to a tertiary care center for mechanical pulmonary thrombectomy.

**Figure 3:**
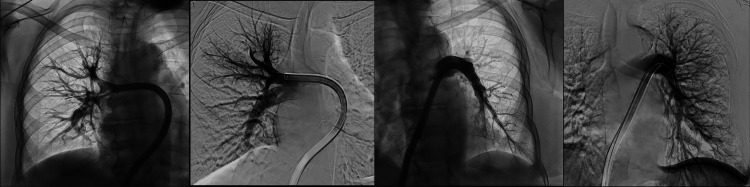
Pulmonary thrombectomy: CT images demonstrating pre-thrombectomy and post-thrombectomy results in the right and left lungs CT, computed tomography.

The patient underwent successful thrombectomy (Figure 3) with improvement in her hemodynamics and resolution of her abdominal pain. She was started on oral anticoagulation and was discharged with hematology follow-up to guide long-term anticoagulation in the setting of underlying malignancy.

## Discussion

Diagnostic value of abdominal CT in atypical PE presentations 

Abdominal pain is a rare manifestation of PE but has been previously described in the literature [2,3]. A retrospective analysis found that in a series of 42 patients with angiographically proven PE, only 9.5% presented with abdominal pain [4]. It is hypothesized that hepatic congestion from right ventricular strain, mesenteric hypoperfusion, and diaphragmatic or pleural irritation may provoke abdominal pain in these cases [5]. Careful and purposeful abdominal diagnostic assessments are critical to avoid unnecessary surgical interventions.

Modern cross-sectional CT scans of the abdomen/pelvis often include the lung bases and have the ability to detect segmental and subsegmental branch PE's, even without being a designated angiographic study [6]. Lim et al. found that among 18 patients with confirmed PE, 7 (38.9%) had emboli observed on a previous CT abdomen study within three months of the initial diagnosis [7]. When evaluating unexplained abdominal syndrome, it is important to review any pulmonary arteries visible on CT, as incidental emboli burden may prove to be clinically significant.

MM and MG: clinical significance in atypical PE

Both MM and MG represent inflammatory disease states; however, the strength of evidence and underlying mechanisms differ substantially between the two conditions.

MM is a well-established hypercoagulable disease with a markedly increased risk of venous thromboembolism [8]. PE has previously been documented as the initial manifestation of MM and may precede classic features such as bone pain, anemia, or renal dysfunction [9]. This elevated risk is multifactorial and includes chronic systemic inflammation, elevated immunoglobulin levels, impaired fibrinolysis, acquired resistance to activated protein C, and endothelial dysfunction [10].

In contrast, the association between MG and PE is less well-defined, supported primarily by isolated case reports rather than epidemiologic data [11]. Proposed mechanisms include autoimmune-mediated inflammation and endothelial injury, which may predispose to thrombosis even in the absence of traditional risk factors.

## Conclusions

This case highlights a rare but clinically important presentation of high-risk PE manifesting predominantly as acute gastrointestinal pathology. In the absence of classic cardiopulmonary complaints, PE may be easily overlooked, especially in elderly patients with multiple comorbidities. Our patient’s presentation underscores the importance of maintaining a high index of suspicion for PE in patients with unexplained abdominal pain, particularly in the setting of known hypercoagulable conditions such as malignancy. Careful review of the lung bases on abdominal CT imaging can provide a critical diagnostic clue and expedite life-saving intervention. Early recognition and prompt multidisciplinary management are essential to reduce morbidity and mortality associated with atypical and high-risk PE presentations.

## References

[1] Khasin M, Gur I, Evgrafov EV, Toledano K, Zalts R (2023). Clinical presentations of acute pulmonary embolism: a retrospective cohort study. Medicine (Baltimore).

[2] Jolobe OMP (2020). Atypical manifestations of pulmonary embolism. Arch Vas Med.

[3] Rehman H, John E, Parikh P (2016). Pulmonary embolism presenting as abdominal pain: an atypical presentation of a common diagnosis. Case Rep Emerg Med.

[4] Gantner J, Keffeler JE, Derr C (2013). Pulmonary embolism: an abdominal pain masquerader. J Emerg Trauma Shock.

[5] Gorham LW (1961). A study of pulmonary embolism. III. The mechanism of pain; based on a clinicopathological investigation of 100 cases of minor and 100 cases of massive embolism of the pulmonary artery. Arch Intern Med.

[6] Klok FA, Huisman MV (2017). Management of incidental pulmonary embolism. Eur Respir J.

[7] Lim KY, Kligerman SJ, Lin CT, White CS (2014). Missed pulmonary embolism on abdominal CT. AJR Am J Roentgenol.

[8] Kristinsson SY, Fears TR, Gridley G, Turesson I, Mellqvist UH, Björkholm M, Landgren O (2008). Deep vein thrombosis after monoclonal gammopathy of undetermined significance and multiple myeloma. Blood.

[9] Sen P, Kumar A, Mathews M, Macbruce D, Miller R (2015). Pulmonary embolism: a rare initial manifestation of multiple myeloma. Am J Med Case Rep.

[10] Kim SJ, Kim K, Kim BS (2008). Bortezomib and the increased incidence of herpes zoster in patients with multiple myeloma. Clin Lymphoma Myeloma.

[11] Lin S, Wang Y, Guan W, Shi Y (2018). Pulmonary embolism caused by myasthenia gravis: a case report. Medicine (Baltimore).

